# Natural wax-based edible coatings for preserving postharvest quality of mandarin orange

**DOI:** 10.1016/j.fochx.2025.102302

**Published:** 2025-02-19

**Authors:** L. Susmita Devi, Avik Mukherjee, Shikha Sharma, Vimal Katiyar, Joydeep Dutta, Santosh Kumar

**Affiliations:** a Department of Food Engineering and Technology, Central Institute of Technology Kokrajhar, Kokrajhar, Assam 783370, India; b International Joint Programme in Food Science and Technology, Department of Chemical Engineering, Indian Institute of Technology (IIT) Guwahati, Guwahati, Assam 781039, India; c Functional Materials, Department of Applied Physics, School of Engineering Sciences, KTH Royal Institute of Technology, Hannes Alfvéns väg 12, Stockholm 11419, Sweden

**Keywords:** Carnauba wax, Shellac, Carvacrol, Essential oil, Antimicrobial coating, Shelf-life

## Abstract

Mandarin oranges are susceptible to senescence and decay, primarily due to postharvest quality loss and fungal infections. This study aim to develop edible and active coatings using carnauba and shellac incorporated with carvacrol nanoemulsion (CNE), and to examine the synergistic effects of the coating on the quality parameters and shelf-life of the mandarin oranges during ambient storage. Nanoemulsion of carvacrol with average droplet size of 348.8 nm was prepared, showing a polydispersity index value of 0.211, showed excellent antifungal activities. Five coating formulations were developed and applied on the mandarin oranges. The coating containing 2 %, *v*/v CNE exhibited the lowest weight loss (19.23 %), maintaining highest firmness (12.06 N), and total soluble solids of 16.1°Brix, and a titratable acidity of 1.56 % even after 30 days of storage. The application of the coating on the mandarin oranges-maintained quality up to 30 days doubling the shelf-life in the ambient.

## Highlights

• Carnauba, shellac and carvancrol nanoemulsion based edible coating were developed.
• Coated mandarin fruits had reduced weight loss, maintained firmness, and titratable acidity.
• Coating doubled the shelf-life of mandarin oranges during ambient postharvest storage.
• The developed natural wax-based coating provides an effective preservation technique.

## 1 Introduction

Mandarin orange (*Citrus reticulata* Blanco) is an ancient citrus fruit known for its nutritional richness and bioactive compounds with potential medicinal benefits (Al-Dairi, Pathare, & Al-Yahyai, 2021). Its taste is generally sweeter and stronger than oranges, and has a very thin, pebbled skin with a bitter white mesocarp (Kanwal & Tahir, 2018). According to the Statista 2024 report, mandarin production in India reached over six million metric tons, that is the highest among citrus fruits produced in the country (Keelery, 2023). The substantial production increase has significantly boosted exports of mandarin necessitating the implementation of new strategies to extend shelf-life of the fruits. Mandarins are highly perishable with a postharvest shelf life of only 1–2 weeks, and their quality often deteriorates due to senescence and fungal spoilage apart from physical damages from improper storage (Strano et al., 2021). Fungal spoilage like green and blue decays caused by *Penicillium digitatum* and *Penicillium italicum*, respectively, can lead up to 60 % product losses during the postharvest handling, transportation, and marketing, resulting in significant economic losses for growers and suppliers (Devi, Mukherjee, Dutta, & Kumar, 2023). Various methods, such as low-temperature storage, synthetic wax-coating, chemical treatments, and modified atmospheric packaging, have been used for longer preservation of the fruit. Low-temperature and modified atmospheric storage methods are often economically unviable and inconvenient, as well as prolonged storage of citrus fruits at 0–10 °C can cause chilling injury, leading to aging, which results in the shrivelling and collapse of the tissues (Basumatary, Mukherjee, Katiyar, Dutta, & Kumar, 2022). Moreover, synthetic preservatives have adverse impact on consumer perception, health and environmental safety motivating the development of a safe and sustainable postharvest treatment and technologies that prevent losses of the produce (Wigati, Wardana, Tanaka, & Tanaka, 2023). In recent years, edible wax such as carnauba, shellac, beeswax-based coatings have gained considerable attention as an alternative to synthetic wax coatings. Edible coatings serve dual purposes by carrying antimicrobial and antioxidants, biocontrol agents, or a combination of both, and providing a protective barrier from environmental factors (Basumatary, Kumar, Mitra, & Halder, 2023).

Carnauba wax and shellac, extracted from the leaves of the Brazilian palm tree (*Copernicia cerifera*), and from the resinous secretion of the insect *Laccifer lacca*, respectively, have been widely used as edible coatings for fruits due to its edibility and nutraceutical value. Classified under the lipid group and sourced naturally, these waxes also create a barrier to water vapor diffusion and add gloss to fresh fruits which enhances the fruit's color, which is easily evaluated by consumers, along with the extension of shelf-life and maintaining fruit quality during storage (J. G. Oliveira Filho et al., 2022). Coatings developed from carnauba wax and shellac, either alone or combined with active agents, have potential for application on various fruits and vegetables, such as tomato, papaya, and mango delaying ripening, reducing weight loss, decreasing the occurrence of diseases, and enhancing visual appeal of the coated fruits (Miranda et al., 2022; J. G. Oliveira Filho, Silva, et al., 2022). Plant-derived essential oils (EOs) are rich in biologically active compounds that possess antimicrobial and antioxidant features. However, as plant-derived essential oils are highly volatile compounds, their functional activities diminish quickly after application on fruits and vegetables. A recent study found that wax-based coatings combined with essential oils or their compounds can slow its release and improve stability of essential oils, thus same effectiveness of EOs can be achieved at lower concentration in the formulations (Susmita Devi, Kalita, Mukherjee, & Kumar, 2022). Carvacrol, a compound present in oregano essential oil, is recognized for its effective antimicrobial and antioxidant properties (Ochoa-Velasco et al., 2021). However, carvacrol face challenges such as instability, poor solubility, altering flavour, and is susceptible to degradation upon exposure to light, oxygen, humidity, or higher temperatures, which limits their use as antifungal, antibacterial and antioxidant agents. The preparation of oil/water nano-emulsions addresses these limitations and provides high effectiveness with sustained release of the active substances (Devi, Das, Dutta, & Kumar, 2024). In this study we present the development of edible coatings for mandarin oranges using carnauba wax and shellac to extend its shelf-life as well as to maintain quality and freshness. Furthermore, effects of incorporation of carvacrol nanoemulsions into the prepared carnauba wax and shellac-based coatings on the coating formulations, as well as on the shelf-life of coated fruits were evaluated and discussed.

## 2 Material and methods

### 2.1 Collection of materials

Carnauba wax No-1 yellow and carvacrol (purity ≥99 %) were obtained from Sigma-Aldrich, India, whereas Tween 80 was purchased from Sisco Research Laboratories Pvt. Ltd., Maharashtra, India. Shellac flake, Mueller Hinton Agar (MHA) media, and potato dextrose agar (PDA) were sourced from HiMedia Pvt. Ltd., Mumbai, India, while sodium hypochlorite (NaOCl) was procured from Avantor Performance Materials, Maharashtra, India. Mandarin oranges (*Citrus reticulata* Blanco) of similar size, color, maturity, and those without any visible damages, were collected from Karigaon, Assam, India. The antifungal studies were performed against *Saccharomyces cerevisiae, Rhizopus stolonifera, Penicillium italicum* and *Aspergillus niger*, and the fungal strains were sourced from the Microbial Type Culture Collection (MTCC) and Gene Bank of CSIR-Institute of Microbial Technology, Chandigarh India.

### 2.2 Preparation of carvacrol nanoemulsions (CNEs)

Carvacrol nanoemulsions (CNEs) were prepared using a two-step process i.e., spontaneous emulsification followed by ultrasonication, as detailed by Ragab et al. (2022) with minor modifications (Ragab et al., 2022). Briefly, Tween 80, a non-ionic surfactant, was added to 2.5 % carvacrol in four different concentrations (2.5 %, 5 %, 7.5 %, and 10 % *v*/v). This mixture was then added dropwise to double-distilled water with continuous stirring on a magnetic stirrer (REMI group, Mumbai, India) to form a coarse emulsion of carvacrol. Ultrasonication of the emulsion at 2 °C for 15 min was then performed by a probe-type sonicator (Vibra Cell VCX-750, Sonics & Materials, Inc., Newton, USA) with a 13 mm diameter probe, operating at a high frequency of 20 kHz of 40 % amplitude and a power output of 750 W. Ice cubes were used to maintain the reaction mixture at 25 °C during sonication, and finally four different nanoemulsion samples were obtained each with a different concentration of Tween 80 i.e., 2.5 %, 5 %, 7.5 %, and 10 %, which were termed as CNE-1, CNE-2, CNE-3, and CNE-4 respectively (Fig. S-1). The prepared CNEs were stored in airtight glass bottles in ambient conditions until characterization and future use.

### 2.3 Characterizations of CNEs

Dynamic light scattering (DLS) (Litesizer 500, Anton Paar) was used to measure polydispersity index (PDI) and mean particle size of the prepared CNEs after their dilution (20 % *v*/v) in deionized water to minimize multiple scattering effects. Each sample was measured 3 times, and the averages as well as standard deviations were recorded. The PDI values between 0.1 and 0.25 indicate a narrow distribution, and values above 0.5 suggest a broad distribution, as reported by Mohammed, Muhialdin, and Meor Hussin (2020) (Mohammed et al., 2020). A capillary cuvette equipped with an electrode (Litesizer DLS 500) determined the zeta potential values of the diluted (20 % v/v) CNEs that were used to determine the charge of the droplets. Zeta potential provides an indicator of the nanoemulsion's stability, and a zeta potential value of at least ±30 mV ensures nanoemulsion stability, which indicate sufficient electrostatic repulsion to prevent particle coalescence (Mohammed et al., 2020). A viscometer (Brookfield Ametek, USA) with a cylindrical spindle (LV5) operating at a speed of 80 rpm was used to determine viscosity of the prepared CNEs under ambient conditions, whereas a FTIR spectrometer (Spectrum Two PerkinElmer, Inc., Waltham, MA) was used for recording the FTIR spectra within the wave-number range of 4000–500 cm^−1^ at a resolution of 4 cm^−1^.

To check the physical stability, the CNEs were centrifuged at 10,000 rpm for 20 min, and 50 mL of the CNEs were stored at 25 ± 1 °C, 37 ± 1 °C and 60 ± 1 °C for one month, during which the samples were visually monitored for any obvious phase separation, creaming or flocculation. For thermal stability studies, 50 mL of the CNEs were heated to 80 °C, 90 °C, and 100 °C for 30 min, and the visual changes in the nanoemulsions were recorded. The antifungal activities of the prepared CNEs were evaluated using the modified agar well diffusion method. To assess antifungal activities, *Saccharomyces cerevisiae, Rhizopus stolonifera, Penicillium italicum* and *Aspergillus niger* were cultured on nutrient agar at 25–30 °C for 48 h. Subsequently, 200 μL of each of the fungal suspension was spread on potato dextrose agar (PDA). Wells were then created in the agar, each well was filled with 200 μL of the prepared CNEs, and the zone of inhibition (ZoI) was measured by an electronic digital calliper (Hitech Survey Tools Pvt. Ltd., Haryana, India) after incubation of the PDA plates at 25 °C for 24 h.

### 2.4 Preparation of coating formulations

Initially, 1:1 mixture of shellac and carnauba wax, each at a concentration of 3 % (*w*/*v*), was dissolved in an alkaline solution (0.5 % ammonium hydroxide) at 95 °C with continuous stirring at 3000 rpm using a magnetic stirrer (REMI, Mumbai, India), with oleic acid serving as an emulsifier. Subsequently, carvacrol nanoemulsion (CNE-3) was added into this admixture at concentrations of 2 % and 4 % (*v*/v). Additionally, separate solutions containing only 3 % (w/v) of carnauba wax and 3 % (w/v) of shellac were prepared as additional coating formulations. Consequently, five different coating formulations, denoted as CF1, CF2, CF3, CF4, and CF5, were formulated as summarized in Table S-1.

### 2.5 Characterization of the prepared coating formulations

The polydispersity index (PDI), particle size, zeta potential, storage stability, viscosity, and FTIR of the coating formulations were evaluated using the same methods applied for characterizing CNEs, as mentioned in section 2.3. Additionally, the antifungal activity of the coating formulations were tested against the same fungal pathogens, *Saccharomyces cerevisiae, Rhizopus stolonifera, Penicillium italicum and Aspergillus niger*, using the well diffusion method as discussed in section 2.3, and outlined in our previous study (Das, Devi, Dutta, & Kumar, 2024).

### 2.6 Application of the coating on mandarin orange

**Fig. 1:**
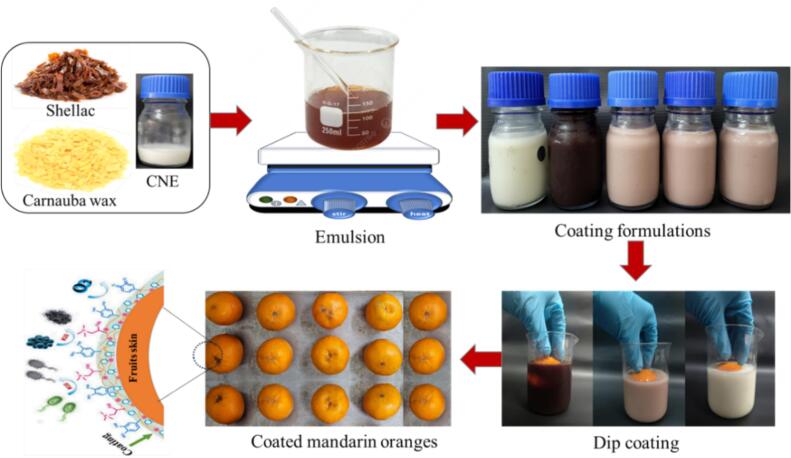
Schematic representation of the preparation and application of the coating formulation.

The mandarin oranges were washed with water to remove surface contamination and then sanitized by immersing them in sodium hypochlorite solution (2 % *v*/v) for 2 min. A collection of selected 150 mandarins were classified into six groups, and each group of mandarin was dip-coated separately with CF1, CF2, CF3, CF4, and CF5 by dipping into the coating formulation for 30 s, followed by the removal of the mandarin from the coating formulation, placing them on a tray to remove the excess coating formulation, followed by storage of the treated fruits indoors in ambient conditions (25 °C and 70 % RH) for 30 days. The untreated 6th group of mandarin was used as the control. During storage, the treated fruits were analyzed for weight loss, firmness, total soluble solids (TSS), titratable acid (TA), decay index, sensory analysis including appearance at every 5 days interval. The preparation of the coating formulations and their application on mandarin are summarized in Fig. 1.

### 2.7 Determination of quality of the coated mandarin orange

#### 2.7.1 Weight loss, firmness, TSS and TA

The weight of the mandarin was recorded at every 5 days interval using an electronic weighing scale, whereas firmness of the mandarin samples was measured using a texture analyzer (TA. XT Plus, Stable MicroSystem Ltd., UK) equipped with a 6 mm diameter cylindrical probe, operating at a velocity of 10 mm/s. Firmness of each fruit sample was tested at three different locations (below, upper, and either right or left), and the maximum force in Newton (N) was recorded using the force vs. time curve. Determination of titratable acidity (TA, g/L of citric acid), and total soluble solid (TSS, °Brix) were carried out using the method described by Barkaoui et al. (2020) (Barkaoui et al., 2020). 10 g of mandarin pulp was homogenized, and then diluted with 50 mL water. The diluted samples were placed in a water bath at 80 °C for 30 min, followed by filtration. The titratable acidity (TA) of the prepared mandarin juice was measured by titration with 0.1 mol/L NaOH solution using phenolphthalein indicator. TSS contents of the mandarin were measured by a digital refractometer (Atago, Tokyo, Japan) by placing one drop of mandarin homogenate on the refractometer.

#### 2.7.2 Decay index (DI)

Visual assessment was used to measure the degree of microbial decay on each fruit surface using the following scale; 0 = healthy fruit, 1 = one very small lesion (1 % ∼ 20 % infection), 2 = few lesions or 21 % ∼ 40 % of fruit surface infected, 3 = several lesions or 41 % ∼ 60 % of fruit surface infected, 4 = 61 % ∼ 80 % of fruit surface infected, and 5 = spores were formed on fruits surface or more than 81 % of fruit surface was infected. The percentage decay was calculated by the following equation.

$$\text{Decay index}\;\left(\%\right)=\sum \left(d\times f\right)/\left(D\times N\right)\times 100$$

where, *d* is the category of fruit, fis its frequency, D is the category of highest decay intensity on an empirical scale and N is the total number of fruits.

#### 2.7.3 Yeasts and molds count

The microbiological determination of the treated fruits was conducted to assess the total yeast and mold counts of the juices using the pour plate technique. 30 g of the mandarin juice was diluted tenfold with peptone saline solution. After homogenizing in a stomacher, serial dilutions of the homogenate were prepared, and spread-plated on PDA followed by incubation at 25 °C for 72 h. after the incubation, the colony counts were taken, and expressed as log CFU/g.

#### 2.7.4 Sensory evaluation

The sensory evaluation of the fruits were conducted by adopting the protocol of Zhou et al. (2023) with slight variations (Zhou et al., 2023). 10 trained panels, consisting of students and faculties between 20 and 40 years of age were selected from the Central Institute of Technology Kokrajhar for the sensory assessments. The order of the samples were randomized for each panellist, who visually evaluated the fruits every 5 days interval during storage by evaluating attributes such as appearance, color, brightness, and intention to buy the products. A 9-point hedonic scale was used to evaluate relative approval scores, with 9 indicating “like extremely”, 5 indicating “neither like nor dislike”, and 1 indicating “dislike extremely”.

### 2.8 Statistical analysis

All the analyses were carried out in triplicates, and the results were expressed as the mean ± standard deviation. To compare the means between different treatments, a one-way analysis of variance (ANOVA) with the least significant difference (LSD) test was conductedusing OriginPro statistics software, and statistically significant differences were considered at *P* < 0.05.

## 3 Results and discussion

### 3.1 Characteristics of the prepared CNEs

#### 3.1.1 Viscosity and stability

The impact of varying concentrations of Tween 80 (2.5 %, 5 %, 7.5 %, and 10 %, *v*/v) on the viscosity and storage stability of CNEs are provided in detail in Table 1. The nanoemulsion with the lowest concentration of Tween 80 (2.5 %) are least viscous (5.2 cP), whereas the emulsion prepared with 10 % Tween 80 concentration exhibited the highest viscosity (10.53 cP). The increase in viscosity at higher surfactant concentrations occur due to the formation of a thick, charged interfacial layer that minimizes droplet aggregation and, consequently, maintains high viscosity (Kaur, Singh, & Sharma, 2021). These findings are in agreement with a previous study, where the viscosity of the nanoemulsion was reported to increase from 5 to 105 mPa.s, when concentration of surfactant (Tween 80) was raised from 5 to 30 % (Das et al., 2024). Similarly, in a neem seed oil-based nanoemulsion, as the oil-to-surfactant ratio increased from 1:1 to 1:3, the viscosity of the nanoemulsions rose from 8.01 to 60.02 cP, indicating that higher surfactant concentrations result in greater viscosity (Devi et al., 2023). The storage stability of the prepared CNEs were evaluated through centrifugation, thermal stability tests, and visual inspection during the ambient storage. All the CNEs remained stable maintaining their milky appearance without any signs of creaming or phase separation during both the stress test and storage period. The results were consistent with similar studies reported by Das et al. (2024), in which nanoemulsion samples were found to be stable, exhibiting no signs of phase separation during both stress and storage tests (Devi et al., 2023).

**Table 1:** Characteristics of as-prepared CNEs.

Emulsion code	Viscosity (cP)	Storage stability	PDI	*Z*-average (nm)	Zeta potential (mV)
CNE-1	5.2 ± 0.1^a^	Stable	0.18	217.0	−30.6
CNE-2	5.8 ± 0.03^a^	Stable	0.202	255.9	−97.0
CNE-3	6.76 ± 0.65^a^	Stable	0.211	348.8	−59.5
CNE-4	10.53 ± 1^b^	Stable	0.23	750.7	2.0

[CNE; Carvacrol nanoemulsion with different concentration of Tween 80, i.e., 2.5, 5.0, 7.5, and 10 %, (*v*/v)].

#### 3.1.2 Polydispersity index (PDI) and droplet size

**Fig. 2:**
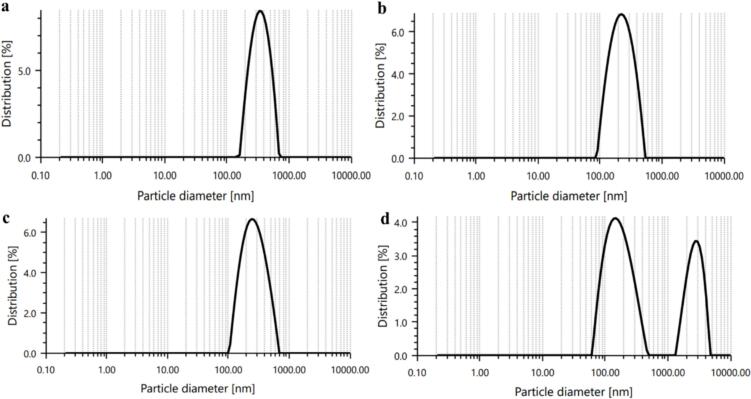
Effect of tween 80 concentration in the nanoemulsions on particle size distribution of a) CNE-1, b) CNE-2, c) CNE-3, and d) CNE-4.

The polydispersity index and mean droplet diameter (Z-averages) of the CNEs are summarized in Table 1 and Fig. 2. The PDI value of the developed nanoemulsions increased slightly from 0.18 to 0.23 as the concentration of Tween 80 was raised. The Z-averages of the nanoemulsions varied between 217 and 750.7 nm, with the size generally increasing alongside higher concentrations of Tween 80, however, nanoemulsion with 5 % Tween 80 (CNE-2) exhibited a larger droplet size with a mean diameter of 348.8 nm, compared to CNE-3 (255.9 nm). This can be attributed to the larger droplets not being fully disrupted in a single pass, possibly due to the higher shear rate generated in the reaction chamber (Wan, Zhong, Schwarz, Chen, & Rao, 2019). The particle size of a nanoemulsion is influenced by factors such as processing conditions, interactions between components, and the adsorption of surfactants onto the oil phase (Wu et al., 2021). The particle size distribution of the nanoemulsions is unimodal with a single peak at droplet sizes of 356.5 nm, 237.3 nm, and 282.2 nm for the CNE-1, CNE-2, and CNE-3 samples, respectively. In contrast, the CNE-4 sample exhibited a bimodal size distribution with two peaks at droplet sizes of 175.65 nm and 275.1 nm. Recent studies by Oliveira Filho et al. (2022) and Mazarei and Rafati (2019) using carvacrol nanoemulsions with Tween 80 and Span 80 as surfactants have reported similar droplet sizes, ranging from 125 to 164 nm and 148 to 151 nm, respectively (Mazarei & Rafati, 2019; Motta Felício et al., 2021). Furthermore, Dantas et al. (2021) conducted a study on carvacrol nanoemulsions, reporting a *Z*-average value of 169.06 nm with a polydispersity index of 0.14 after 90 days of storage, indicating excellent stability of the nanoemulsion (Dantas et al., 2021).

#### 3.1.3 Zeta potential of the CNEs

**Fig. 3:**
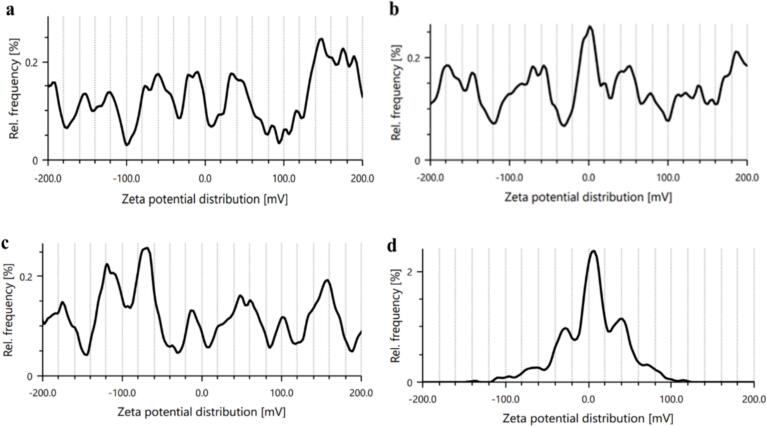
Effects of tween 80 concentration on zeta potential of; a) CNE-1, b) CNE-2, c) CNE-3, and d) CNE-4.

Zeta potential is a key indicator used to assess the electrostatic stabilization of droplets and particles, making it an indirect measure of the physical stability of lipid nanoparticles (Souza, Saez, & Mansur, 2023). The impact of increasing surfactant content on the zeta potential of the formulated nanoemulsion are presented in Table 1 and Fig. 3. The results demonstrate that increasing the concentration of Tween 80 initially raised the zeta potential values from −30.6 mV to −97.0 mV as the surfactant concentration increased from 2.5 % to 5 %. However, as the concentration was further increased to 7.5 %, the zeta potential gradually decreased to −59.5 mV, and a significant drop to 2 mV was observed when the surfactant concentration reached 10 %. This decrease in zeta potential could be attributed to the increased absorption of surfactant on the surface of the oil droplets, leading to a reduction in the charge on the droplet surface (Indra Bhusan & Santosh, 2024). Nanodispersions with zeta potential values ranging between ±30 and ± 60 mV typically exhibits good physical stability in nanoemulsions, reducing the likelihood of flocculation, which is commonly observed when the zeta potential is below 15 mV (Mann, Singh, Athira, Kumar, & Sharma, 2022). Consequently, managing zeta potential can be an effective strategy to enhance the physical stability of nanodispersions containing ionic emulsifiers. However, for nonionic stabilizers, physical stability is primarily influenced by steric stabilization rather than electrostatic interactions. In the absence of a true charge, nonionic surfactants form droplets without an electrical double layer, and emulsion stabilization is achieved through steric hindrance, where the polar, often long, molecular segments prevent aggregation (Sonneville-Aubrun, Simonnet, & L'Alloret, 2004; Souza et al., 2023). Our findings align with previous studies, which also observed zeta potentials of carvacrol nanoemulsions of −59 ± 0.9 mV. Similar results have been reported by many other researchers in surfactant-stabilized nanoemulsions, which also demonstrated the adsorption of surfactant (Tween 80) at the oil-water interface (Yang, Leser, Sher, & McClements, 2013).

#### 3.1.4 FTIR of CNEs

**Fig. 4:**
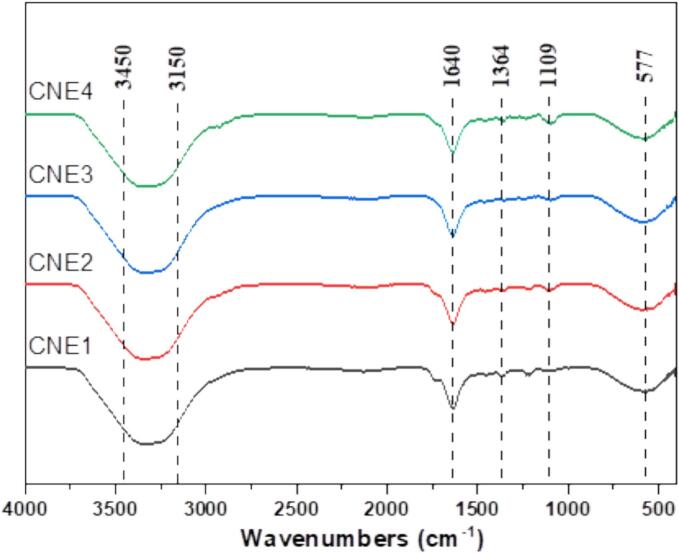
FTIR spectra of the prepared carvacrol nanoemulsions.

The bonding structure differences in CNEs with varying concentrations of Tween 80 were assessed using FTIR spectra, and is shown in Fig. 4. A broad absorption peak observed between 3450 and 3150 cm^−1^, which correspond to the stretching vibrations of -OH groups, indicate inter- and intramolecular hydrogen bonding, as well as physical cross-linking among Tween 80 and the bioactive compounds. A sharp peak at 1640 cm^−1^ reflects the stretching vibrations of C=O groups (amide I) across all the nanoemulsions, and the peak intensity gradually decreased for emulsions prepared with higher concentration of the surfactant (from 2.5 % to 10 %) in the CNEs. The peak at 1364 cm^−1^ corresponds to CH_3_, while the peak around 1109 cm^−1^ is attributed to C—O bending vibrations, suggesting the presence of esters in carvacrol. The characteristic peaks of the CNEs were similar to those reported for thyme essential oil nanoemulsions (Liu & Liu, 2020), and carvacrol nanogels (Sanei-Dehkordi, Hatami, Zarenezhad, Montaseri, & Osanloo, 2022).

#### 3.1.5 Antifungal activities of CNEs

**Fig. 5:**
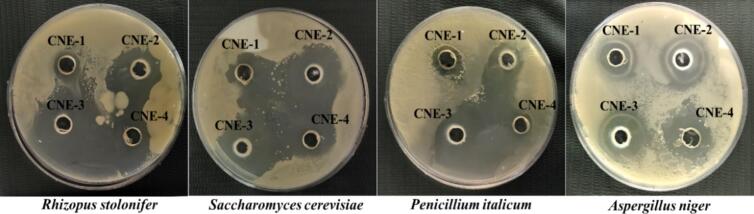
Antifungal effect of CNEs against *R. stolobifer, S. cerevisiae, P. italicum* and *A. niger.* Showing the zone of inhibition.

The in vitro antifungal activities of CNEs against the four common fungal strains; *Rhizopus stolonifer, Saccharomyces cerevisiae, Penicillium italicum, and Aspergillus niger* are shown in Fig. 5**,** and Table S-2. CNE-1, containing the lowest concentration of tween 80, exhibited the smallest zones of inhibition 13 mm, 16.5 mm, 14.75 mm, and 18 mm, respectively against all the four fungal strain indicating limited antifungal effectiveness. In contrast, CNE-3 displayed the strongest antifungal activity, with larger inhibition zones, demonstrating its effectiveness in suppressing fungal growth against *Rhizopus stolonifera* (23.67 mm) and *Penicillium italicum* (21.17 mm). CNE-2 showed the highest inhibition against *Saccharomyces cerevisiae* (24.34 mm), and CNE-4 was most effective against *Aspergillus niger* (23.5 mm), highlighting the superior antifungal properties of higher concentrations of carvacrol nanoemulsions. The pendant hydroxyl groups in the coating materials are likely responsible for the observed antifungal effect of the developed coatings. It has been suggested that interactions between the hydroxyl groups and membrane components result in pore formation on the cellular membrane, leading to leakage and ultimately causing cell death (Fei, Leyva-Gutierrez, Wan, & Wang, 2021). In line with these findings, Das et al. (2024) reported that essential oil nanoemulsions demonstrated strong antifungal activity against *Saccharomyces cerevisiae* and *Rhizopus stolonifer*, with inhibition zones of 35.56 mm and 37.59 mm, respectively (Das et al., 2024). A similar study by Fonseca et al. (2021) also reported that carvacrol produced inhibition zones against Penicillium species and Aspergillus flavus, measuring 24.36 mm and 28.74 mm, respectively. These findings suggest that carvacrol exhibits strong antifungal activity (Fonseca et al., 2021).

### 3.2 Characteristics of the developed coating formulation

#### 3.2.1 pH, viscosity, and storage stability of the coating formulations

The characteristics of the prepared coating formulations, such as pH, viscosity, and storage stability are summarized in Table 2. Viscosity is a crucial parameter that affects the final thickness of the coating, influencing several properties including water vapor permeability (Miranda, Ribeiro, et al., 2022). The coating formulation containing only carnauba wax (CF1) exhibited a slightly higher viscosity of 9.42 cP compared to the formulation with only shellac wax (CF2), which had the lowest viscosity of 6.36 cP. Meanwhile, the formulation combining both carnauba and shellac waxes (CF3) resulted in an even more viscous solution, with a viscosity value of 10.57 cP. In contrast, formulations incorporating CNE-3 at concentrations of 2 % (CF4) and 4 % (CF5) showed the highest viscosity values, measuring 12.07 cP and 12.58 cP, respectively. These results are consistent with the findings of Miranda, Ribeiro, et al. (2022) and Devi et al. (2023), who investigated the effects of physicochemical characteristics of carnauba wax emulsion-based edible coating formulations (Devi et al., 2023; Miranda, Ribeiro, et al., 2022). The pH of the coating formulations ranged from 5.93 to 6.33, showing no significant differences between the formulations. All the coating formulations displayed slight instability, with some sedimentation observed during both the stress test and the 30-day storage period, however, CF2 remained stable during ambient storage. A similar stress test was conducted by Devi et al. (2023) on wax-based coating solutions, where the formulations remained stable without any phase separation during both the stress test and storage period, however, after 60 days of ambient storage, all the coating formulations exhibited some sedimentation (Devi et al., 2023).

**Table 2:** Physicochemical characteristics of the prepared coating formulations.

Coating formulation code	Viscosity	Storage stability	pH
CF1	9.42 ± 0.39^a^	Slightly unstable	5.93 ± 0.08^a^
CF2	6.36 ± 0.32^b^	Stable	6.17 ± 0.12^a^
CF3	10.57 ± 0.25^a^	Slightly unstable	6.26 ± 0.12^a^
CF4	12.07 ± 0.02^c^	Slightly unstable	6.33 ± 0.28^a^
CF5	12.58 ± 0.41^c^	Slightly unstable	6.33 ± 0.12^a^

Values are means ± standard deviations of triplicate determinations and the superscript letters indicate that they are significantly different (*p* < 0.05). [CF1 (3 % carnauba wax); CF2 (3 % shellac wax); CF3 (3 % carnauba +3 % shellac); CF4 (3 % carnauba +3 % shellac +2 % CNE-3); and CF5 (3 % carnauba +3 % shellac +4 % CNE-3)].

#### 3.2.2 Antifungal activities of the coating formulations

**Fig. 6:**
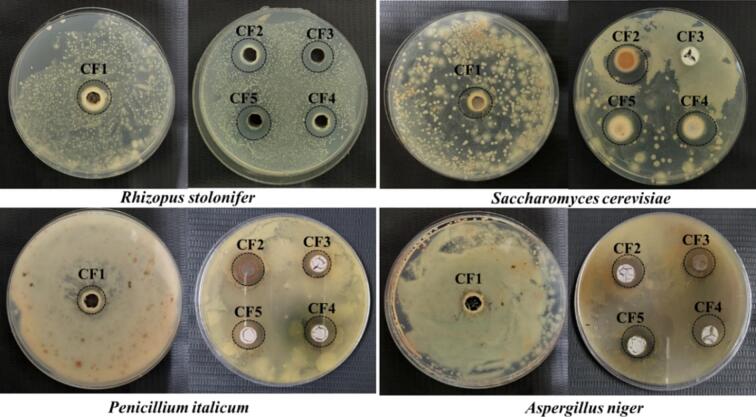
Antifungal effect of the prepared coating formulations against *Rhizopus stolobifer, Saccharomyces cerevisiae, Penicillium italicum* and *Aspergillus niger* showing the zone of inhibition.

The inhibitory effects of the developed coating formulations against *R. stolonifera, S. cerevisiae, P. italicum,* and *A. niger* are shown in Fig. 6, and Table S-3 that exhibited antifungal activity against all the four tested fungi. CF1 showed the smallest inhibition zone of 12.84 mm against *R. stolonifera*, while CF2 had the largest zone of inhibition of 15.06 mm, followed by CF5 (14.6 mm), CF4 (14.23 mm), and CF3 (14.03 mm). Against *S. cerevisiae*, CF2 displayed the highest inhibition zone of 14.6 mm, followed by CF5 (14.21 mm), CF4 (13.7 mm), and CF1 (13.47 mm), while CF3 showed no inhibition. For *P. italicum*, CF4 had the largest inhibition zone, whereas CF1 had the smallest zone of 13.5 mm. Finally, against *A. niger*, CF1 showed no inhibition, while CF2, CF5, and CF4 exhibited almost similar inhibition zones of 14.35 mm, 14.33 mm, and 14.26 mm, respectively, while CF3 had the smallest zone of inhibition of 13.23 mm. Consistent with these findings, Fei et al. (2021) reported that shellac and carnauba wax emulsion exhibited effective antifungal activities against *A. niger,* with inhibition zones of 4.90 mm and 4.35 mm, respectively (Fei et al., 2021). A similar observation was made by Won and Min (2018), where the incorporation of grapefruit seed extract into carnauba wax coating resulted in effective antifungal activity against *P. italicum* (Won & Min, 2018).

### 3.3 Effects of the developed coatings on mandarin oranges

#### 3.3.1 Weight loss and firmness

**Fig. 7:**
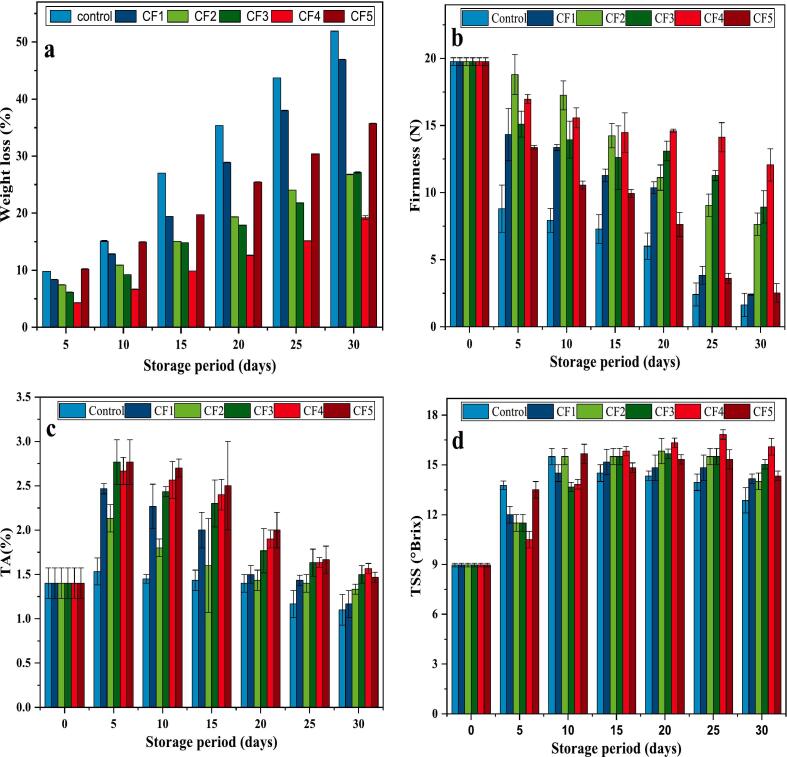
Effects of the coatings on mandarin oranges fruit samples; a) weight loss, b) firmness, c) TA, and d) TSS.

The effects of coating on weight loss and firmness of the coated fruit samples during 30-day storage are presented in Fig. 7a and b. Weight loss is a critical factor affecting overall quality, and marketability (Lufu, Ambaw, & Opara, 2020). The results showed that weight loss in all the fruit samples significantly increased over time, however, the control group experienced a notably higher weight loss of 51.93 % compared to all the other coated fruits after 30 days of storage. The greatest reduction in weight loss was observed in fruits treated with CF4, with 19.23 % weight loss, followed by CF2 (26.80 %), CF3 (27.12 %), CF5 (35.35 %), and CF1 (46.94 %). These results suggest that the hydrophobicity of natural wax was enhanced by the addition of CNE to the coating, because of reduced availability of hydroxyl groups for interaction with water molecules, and cross-linking effect created by the interaction between the wax and CNE. The findings align with the report of Khorram and Ramezanian (2021), where lipid coatings containing essential oils were shown to reduce weight loss by 52 % in coated ‘Thomson navel’ oranges (Khorram & Ramezanian, 2021). Fruits firmness is a crucial parameter linked to maturity and juiciness, that influences consumer perception and postharvest storage quality. The fruit's firmness rapidly deteriorated during storage, however, the rate of decline varied depending on the treatments (Fig. 7b). The control group of mandarin oranges showed the fastest deterioration having the lowest firmness value of 1.63 N, while CF4 treated fruits maintained the highest firmness of 12.06 N by the end of 30 days storage, followed by CF3 (8.93 N), CF2 (7.64 N), CF5 (2.51 N), and CF1 (2.39 N). The obtained results show that the developed coatings effectively maintained the firmness of the fruit's that could be due to retention of cell turgor pressure, and slowing down ripening (Al-Dairi et al., 2021). These results are consistent to the findings of Yan, Zhang, Hu, Deng, and Ritenour (2020), in which coating of shellac wax and carvacrol significantly slowed fruit softening and retained 13 % more firmness compared to the control after 8-weeks of storage (Yan et al., 2020). Miranda et al. (2022) also reported a reduction in loss of firmness in papaya fruits treated with nano- and micro-sized carnauba wax emulsion-based coatings that incorporated ginger essential oil and hydroxypropyl methylcellulose (Miranda, Sun, et al., 2022).

#### 3.3.2 Titratable acidity (TA) and total soluble solids (TSS)

Titratable acidity, measures the total acid concentration in food, and the results in this study are shown in Fig. 7c. The TA of the control and coated mandarin oranges showed a gradual increase during the first 10 days of storage, followed by a consistent reduction until the end of the 30 days storage, when the coated mandarin oranges exhibited higher levels of TA compared to the uncoated control (1.1 %). Among the coated samples, CF4 coated fruits had the highest TA value of 1.4 %, followed by CF3 with 1.4 %, CF5 with 1.36 %, CF2 with 1.33 %, and CF1 with 1.16 %. The organic acids in fruits are metabolised during respiration and ripening, leading to a gradual decline in titratable acidity over time (Zarbakhsh, Kazemzadeh-Beneh, & Rastegar, 2020). Therefore, by applying shellac and carnauba wax as an edible coating on mandarin oranges proved to be effective in maintaining TA. Similar results were also reported by other researchers by applying lipid-based composite coatings on Kaji lemons (Das et al., 2024) and strawberries (J. G. d. Oliveira Filho, Silva, et al., 2022). TSS consist primarily of total sugars, along with small amounts of organic compounds, soluble proteins, and amino acids, and the results of TSS in this study are presented in Fig. 7d. Both the coated and control mandarin showed an increase in TSS values during the initial days of storage, with a slight decline during the subsequent storage. Among the samples, CF4 had the highest TSS (16.1°Brix), while the control fruits had the lowest (12.86°Brix) at the end of 30 days storage. This rise in TSS could be attributed to sugar formation as acids and starch are utilized for metabolic activities, leading to moisture loss and the degradation of cell-wall polysaccharides, which ultimately increases TSS (Hajebi Seyed, Rastegar, & Faramarzi, 2021). The applied coating regulates exchange of gases between the coated fruit and its surroundings, adjusting the internal atmosphere and reducing respiration. As a result, the coated samples had higher TSS compared to the uncoated fruits. In a similar study, Chen, Jiang, Zeng, Huo, and Li (2020) found that applying carnauba wax and 1-methylcyclopropene coatings to apples helped retain significantly higher TSS values over the course of storage compared to uncoated fruits (Chen et al., 2020). Furthermore, Das et al. (2024) reported a consistent increase in TSS levels in wax-coated lemons throughout the storage, indicating better retention of sweetness of the coated fruits. This suggests that the wax-based coating was more effective in maintaining TSS content compared to the control (Das et al., 2024).

#### 3.3.3 Decay index of the coated fruits

**Fig. 8:**
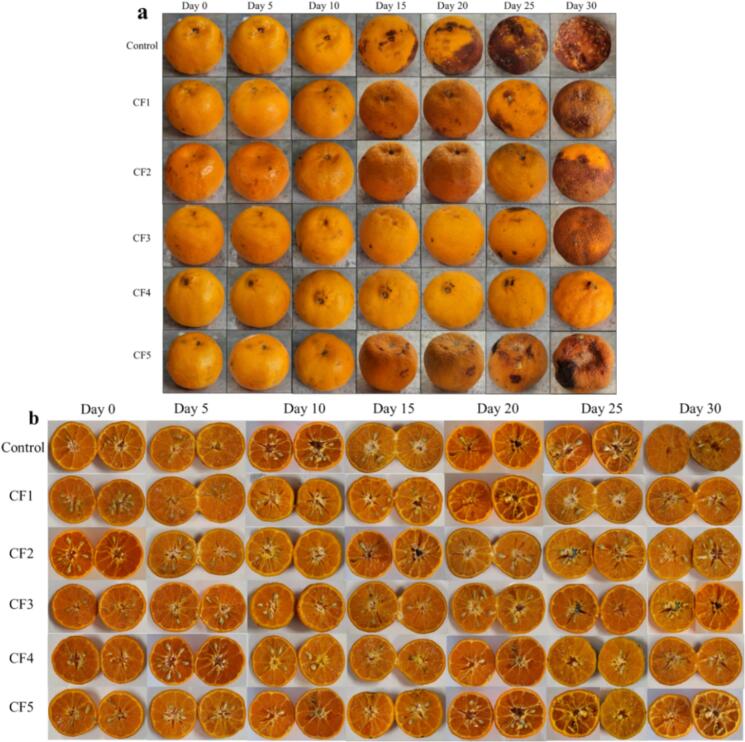
The visual appearance of the coated mandarin oranges during 30 days storage at ambient conditions.

The decay indices of both treated and untreated mandarin oranges are presented in Table S-4, and the visual appearance of whole and cut mandarin oranges are displayed in Fig. 8. The obtained results show that after 14 days of storage, the control fruits exhibit a decay index of 2.83 %, while CF5-treated fruits showed 1.33 % decay, whereas fruits treated with CF1, CF2, CF3, and CF4 showed no signs of decay. However, decay began to appear after 20 days of storage, and increased consistently over the 30-day period. The decay index is typically linked to weight loss, but fungal colonization can also deteriorate the quality of mandarin oranges (Peralta-Ruiz et al., 2020). By the end of the 30-day storage, the control fruits had the highest decay incidence of 4.96 %, while the CF4 treated mandarins showed the lowest decay index at just 1.13 %, outperforming other treatments in preserving the fruit quality. The combined effects of the shellac and carnauba wax coating incorporated with active agents significantly reduced fungal decay and microbial growth. Consequently, it can be concluded that the optimal concentration of CNE (2 %, *v*/v) in the coating resulted in significantly lowering the rate of decay in mandarin oranges. Devi et al. (2023) reported similar findings for carnauba wax coated citrus fruits that exhibited the lowest decay at 2.66 %, whereas the uncoated citrus fruits had the highest decay index at 4.16 % on day 28 of ambient storage (Devi et al., 2023). Several similar studies have also reported on the effectiveness of lipid-based coatings in reducing decay in pomegranates fruits stored for 4 and 8 weeks at 20 °C, and demonstrated significantly lower decay rates, with only 7.5 % decay compared to 35 % in uncoated control fruits (Di Millo et al., 2021).

#### 3.3.4 Yeast and mold count

The impact of various coating treatments on the microbiological properties of mandarin oranges during storage is presented in Table 3, which shows growth of yeasts and molds in both coated and uncoated fruits. It is evident that yeast and mold growth in the control sample began after 10 days, with a microbial count of 2.74 log cfu/g, and in contrast, no growth was observed in the coated samples for up to 15 days. Mandarin fruits treated with CF4 showed no microbial growth for up to 20 days, and had the lowest microbial count of 3.40 log cfu/g even by the end of the 30 days of storage, followed by CF1 (7.43 log cfu/g), CF3 (8.36 log cfu/g), CF2 (9.45 log cfu/g), and CF5 (11.42 log cfu/g). The control samples were fully spoiled by day 20, however all the coated fruits exhibited lower fungal loads compared to the control could be due to antifungal activities of the CNEs incorporated in the shellac and carnauba wax-based coatings. An earlier study reported that the yeast and mold populations in fresh apples initially measured at 1.8 log cfu/g increased significantly to 4.5 log cfu/g in uncoated apples during storage, while yeast and mold populations were effectively inhibited in shellac-coated apples, maintaining levels of 2.1–2.2 log cfu/g compared to the uncoated control fruits under similar storage conditions (Ali, Basu, & Mazumder, 2020).

**Table 3:** Yeast and mold count of the coated and uncoated mandarin oranges during storage.

Treatments	Number of Days (log cfu/g)						
	0	5	10	15	20	25	30
Control	ND	ND	2.74 ± 0.02^a^	3.31 ± 0.008^a^	7.4 ± 0.005^a^	10.40 ± 0.01^a^	**
CF1	ND	ND	ND	2.69 ± 0.01^b^	3.27 ± 0.01^b^	5.48 ± 0.01^b^	7.43 ± 0.01^a^
CF2	ND	ND	ND	2.68 ± 0.03^b^	3.13 ± 0.01^c^	7.31 ± 0.01^c^	9.45 ± 0.008^b^
CF3	ND	ND	ND	2.68 ± 0.006^b^	2.82 ± 0.008^d^	4.42 ± 0.01^d^	8.36 ± 0.008^c^
CF4	ND	ND	ND	ND	ND	2.89 ± 0.01^e^	3.40 ± 0.01^d^
CF5	ND	ND	ND	3.27 ± 0.01^a^	7.43 ± 0.01^a^	10.35 ± 0.008^a^	11.42 ± 0.01^e^

ND: Is represented yeast and mold count not detected. Values are means ± standard deviations of triplicate determinations and the superscript letters indicate that they are significantly different (*p* < 0.05). [Control (uncoated); CF1 (3 % carnauba wax); CF2 (3 % shellac wax); CF3 (3 % carnauba +3 % shellac); CF4 (3 % carnauba +3 % shellac +2 % CNE-3); and CF5 (3 % carnauba +3 % shellac +4 % CNE-3).]

#### 3.3.5 Sensory qualities

Both coated and uncoated mandarin oranges were evaluated for sensory attributes during storage, and the results are presented in Table 4. With respect to fruit appearance, the control sample had the lowest sensory score of 1.66 at the end of the 30-day storage period, which falls below the acceptable threshold of 5 on the hedonic scale. Meanwhile, mandarins coated with CF4 had the highest sensory score of 5.83 even after 30 days of storage, which is considered acceptable. This was followed by CF3, CF2, CF1, and CF5, which had lower sensory scores of 4.83, 4.67, 4.63, and 3, respectively, all falling below the acceptable sensory score threshold. However, all coated mandarins achieved an acceptable sensory score above 5, except for CF5 and the control group, which still scored below the acceptable threshold with scores of 4.83 and 4.33, respectively, on the day 25 of storage. A similar trend was observed in the pulp appearance, color, and intention-to-buy parameters, where the CF4 treated mandarins consistently reported the highest acceptable sensory score of above 5 throughout the storage period. In contrast, the control group and mandarins treated with CF5 showed the lowest sensory scores at the end of the 30-day storage period. In a study, Khorram and Ramezanian (2021) reported that incorporating cinnamon essential oil into shellac coatings for oranges significantly enhanced sensory acceptability, did not compromise fruit quality, and no visible skin damage was observed (Khorram & Ramezanian, 2021). Some authors previously reported that carnauba wax-coated salacca fruits displayed superior sensory attributes, particularly in terms of flavour, compared to uncoated fruits, achieving a hedonic scale score of 6.4 (Phothisuwan, Koomhin, Matan, & Matan, 2021).

**Table 4:** Sensory attributes of coated / treated mandarin oranges during 30 days storage.

Parameters	Days	Treatments					
		Control	CF1	CF2	CF3	CF4	CF5
Fruits appearance	0	8.83 ± 0.16^a^	8.86 ± 0.16^a^	8.83 ± 0.16^a^	8.86 ± 0.16^a^	8.86 ± 0.16^a^	8.86 ± 0.16^a^
	5	8.16 ± 0.16^a^	8.5 ± 0.28^a^	8.33 ± 0.16^a^	8.16 ± 0.16^a^	8.66 ± 0.28^a^	8.5 ± 0.16^a^
	10	7.33 ± 0.16^a^	7.67 ± 0.16^a^	7.33 ± 0.16^a^	7.67 ± 0.16^a^	7.67 ± 0.16^a^	7.33 ± 0.16^a^
	15	6.67 ± 0.16^a^	7.16 ± 0.16^a^	7.33 ± 0.16^a^	7.17 ± 0.16^a^	6.83 ± 0.16^a^	6.66 ± 0.16^a^
	20	6.17 ± 0.16^a^	6.33 ± 0.16^ab^	6.83 ± 0.16^ab^	6.93 ± 0.06^b^	6.43 ± 0.06^ab^	6.16 ± 0.16^a^
	25	4.33 ± 0.16^a^	5.33 ± 0.16^bc^	5.83 ± 0.16^bd^	5.93 ± 0.06^bd^	6.16 ± 0.16^bd^	4.83 ± 0.16^ac^
	30	1.66 ± 0.33^a^	4.63 ± 0.18^b^	4.67 ± 0.16^b^	4.83 ± 0.16^b^	5.83 ± 0.16^b^	3 ± 0.57^a^
Pulp appearance	0	8.2 ± 0.15^a^	8.4 ± 0.1^a^	8.3 ± 0.11^a^	8.33 ± 0.16^a^	8.33 ± 0.16^a^	8.36 ± 0.13^a^
	5	7.83 ± 0.28^a^	7.5 ± 0.32^a^	7.5 ± 0.29^a^	7.4 ± 0.44^a^	7.46 ± 0.28^a^	7.36 ± 0.36^a^
	10	7 ± 0.28^a^	6.67 ± 0.16^a^	6.5 ± 0.44^a^	6.5 ± 0.28^a^	6.5 ± 0.28^a^	6.5 ± 0.28^a^
	15	6.33 ± 0.16^a^	6.12 ± 0.33^a^	6 ± 0.33^a^	6.21 ± 0.16^a^	6.41 ± 0.16^a^	6.16 ± 0.18^a^
	20	5 ± 0.57^a^	5.66 ± 0.16^a^	5.33 ± 0.33^a^	5.66 ± 0.16^a^	5.83 ± 0.16^a^	4.66 ± 0.33^a^
	25	1.33 ± 0.33^a^	2.33 ± 0.33^ab^	4.10 ± 0.16^b^	4.16 ± 0.16^b^	5.66 ± 0.44^c^	1.66 ± 0.33^a^
	30	1.00 ± 0.33^a^	2.25 ± 0.33^ab^	3.01 ± 0.16^b^	3.16 ± 0.16^b^	5.20 ± 0.44^c^	1.36 ± 0.33^a^
Color	0	8.4 ± 0.1^a^	8.6 ± 0.1^a^	8.5 ± 0.23^a^	8.6 ± 0.1^a^	8.53 ± 0.03^a^	8.53 ± 0.03^a^
	5	7.96 ± 0.03^a^	8.36 ± 0.18^a^	8.13 ± 0.18^a^	8.06 ± 0.06^a^	8.33 ± 0.16^a^	8.3 ± 0.15^a^
	10	7.66 ± 0.33^a^	7.63 ± 0.13^a^	7.46 ± 0.26^a^	7.83 ± 0.16^a^	7.8 ± 0.15^a^	7.6 ± 0.1^a^
	15	5.83 ± 0.16^a^	6.34 ± 0.34^ab^	7.16 ± 0.16^bc^	7.17 ± 0.16^bc^	7.34 ± 0.16^c^	5.67 ± 0.17^a^
	20	4.33 ± 0.16^a^	5.16 ± 0.16^b^	6.33 ± 0.16^c^	6.83 ± 0.16^cd^	7.16 ± 0.16^d^	4.16 ± 0.16^a^
	25	1.06 ± 0.06^a^	4.16 ± 0.16^bc^	5.16 ± 0.16^c^	4.83 ± 0.16^b^	6.16 ± 0.16^d^	2.66 ± 0.33^e^
	30	1.03 ± 0.03^a^	2.5 ± 0.5^b^	3.16 ± 0.16^b^	3.33 ± 0.33^b^	5.9 ± 0.20^c^	1.03 ± 0.03^a^
Intention to buy	0	8.83 ± 0.16^a^	8.66 ± 0.16^a^	8.83 ± 0.16^a^	8.66 ± 0.16^a^	8.66 ± 0.16^a^	8.66 ± 0.16^a^
	5	8.16 ± 0.16^a^	8.5 ± 0.28^a^	8.33 ± 0.16^a^	8.16 ± 0.16^a^	8.66 ± 0.16^a^	8.5 ± 0.28^a^
	10	7.33 ± 0.16^a^	7.66 ± 0.16^a^	7.33 ± 0.16^a^	7.66 ± 0.16^a^	7.33 ± 0.16^a^	7.33 ± 0.16^a^
	15	6.66 ± 0.16^a^	7.16 ± 0.16^a^	7.33 ± 0.16^a^	7.16 ± 0.16^a^	6.83 ± 0.16^a^	6.66 ± 0.16^a^
	20	6.16 ± 0.16^a^	6.33 ± 0.16^ab^	6.83 ± 0.16^ab^	6.93 ± 0.06^b^	6.43 ± 0.06^ab^	6.16 ± 0.16^a^
	25	4.34 ± 0.16^a^	5.34 ± 0.16^bc^	5.84 ± 0.16^bd^	5.93 ± 0.06^bd^	6.17 ± 0.16^d^	4.84 ± 0.16^ac^
	30	2.66 ± 0.33^a^	4.67 ± 0.16^b^	4.83 ± 0.16^b^	4.63 ± 0.18^b^	5.83 ± 0.16^b^	3 ± 0.57^a^

Values are means ± standard deviations of triplicate determinations and the superscript letters indicate that they are significantly different (p < 0.05) within treatment, between different durations (days) of storage. [Control (uncoated); CF1 (3 % carnauba wax); CF2 (3 % shellac wax); CF3 (3 % carnauba +3 % shellac); CF4 (3 % carnauba +3 % shellac +2 % CNE-3); and CF5 (3 % carnauba +3 % shellac +4 % CNE-3)].

## 4 Conclusions

The study demonstrated development of carnauba and shellac wax-based edible coatings functionalized with carvancrol nanoemulsion, and their effects of quality preservation and shelf-life improvement of mandarin oranges. The coated mandarin fruits, particularly those treated with the coating formulation containing 2 %, v/v carvacrol nanoemulsion (CF4), had significantly reduced weight loss (19.23 %), maintained firmness (12.06 N), total soluble solids (16.1°Brix) and titratable acidity (1.56 %) during 30 days of ambient storage, compared to what was observed for the uncoated control fruits. The coated fruits also had the highest sensory acceptance, and minimal yeast and mold counts, resulting in reduced decay, and extended shelf-life for up to 30 days, compared to only 15-days for the control. The developed natural wax-based coating provides an effective preservation technique that maintain quality and doubled the shelf-life of mandarin oranges during ambient postharvest storage.

## CRediT authorship contribution statement

**L. Susmita Devi:** Writing – original draft, Methodology, Investigation, Formal analysis, Data curation. **Avik Mukherjee:** Writing – review & editing, Validation, Supervision, Software. **Shikha Sharma:** Writing – original draft, Formal analysis, Data curation. **Vimal Katiyar:** Writing – review & editing, Resources, Conceptualization. **Joydeep Dutta:** Writing – review & editing, Validation, Software. **Santosh Kumar:** Writing – review & editing, Validation, Supervision, Resources, Funding acquisition, Data curation, Conceptualization.

## Declaration of competing interest

The authors declare that they have no known competing financial interests or personal relationships that could have appeared to influence the work reported in this paper.

## Data Availability

Data will be made available on request.

## Appendix A Supplementary data

Natural wax-based edible coatings for preserving postharvest quality of mandarin orange Image 1
