# Supplementary material for: Natural wax-based edible coatings for preserving postharvest quality of mandarin orange
Source: Food Chem X. 2025 Feb 19;26:102302. doi: 10.1016/j.fochx.2025.102302 (PMC11889960; doi:10.1016/j.fochx.2025.102302)
Supplement: Supplementary file 1 — Natural wax-based edible coatings for preserving postharvest quality of mandarin orange [file mmc1.docx]

**Supplementary Data**

**Natural wax-based edible coatings for preserving postharvest quality of mandarin orange**

**L. Susmita Devi^a^, Avik Mukherjee^a^, Shikha Sharma^b^, Vimal Katiyar^b^,**

**Joydeep Dutta^c,^ *, Santosh Kumar^a,^ ***

*^a^Department of Food Engineering and Technology, Central Institute of Technology Kokrajhar, Kokrajhar, Assam-783370, India*

*^b^International Joint Programme in Food Science and Technology, Department of Chemical Engineering, Indian Institute of Technology (IIT) Guwahati, Guwahati, Assam, 781039, India*

*^c^Functional Materials, Department of Applied Physics, School of Engineering Sciences, KTH Royal Institute of Technology, Hannes Alfvéns väg 12, Stockholm 11419, Sweden*

***Corresponding author:**

Prof. Joydeep Dutta; [joydeep@kth.se](mailto:joydeep@kth.se)

Dr. Santosh Kumar**;** [s.kumar@cit.ac.in](mailto:s.kumar@cit.ac.in)

**
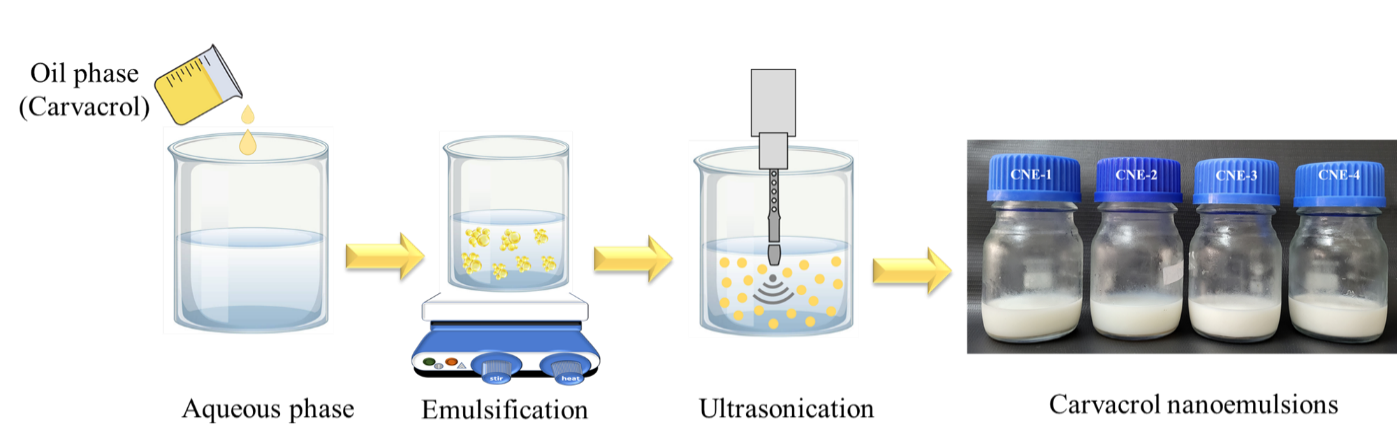
**

**Fig. S-1.** Preparation of carvacrol nanoemulsions (CNEs)

### Table S-1. Composition of the developed coating formulations

| Formulations code | Carnauba wax %, w/v | Shellac %, w/v | CNE-3 %, v/v |
| --- | --- | --- | --- |
| CF1 | 3 | - | - |
| CF2 | - | 3 | - |
| CF3 | 3 | 3 | - |
| CF4 | 3 | 3 | 2 |
| CF5 | 3 | 3 | 4 |

[CF1 (3% carnauba wax); CF2 (3% shellac wax); CF3 (3% carnauba + 3% shellac); CF4 (3% carnauba + 3% shellac + 2% CNE-3); and CF5 (3% carnauba + 3% shellac + 4% CNE-3)]

**Table S-2.** Zone of inhibition of CNEs against selected microbes.

| **Nanoemulsions code** | **Zone of inhibitions (mm)** | | | |
| --- | --- | --- | --- | --- |
|  | ***Rhizopus stolonifer*** | ***Saccharomyces cerevisiae*** | ***Penicillium italicum*** | ***Aspergillus Niger*** |
| **CNE-1** | 13±0.5a | 16.5±0.5a | 14.75±0.25a | 18±0.25a |
| **CNE-2** | 22.67±0.33bd | 24.34±0.34b | 17.84±0.44a | 19.25±0.34b |
| **CNE-3** | 23.67±0.33c | 23.67±0.34b | 21.17±0.60b | 21.33±0.28c |
| **CNE-4** | 21.34±0.33d | 21±0.57c | 17.16±0.72a | 23.5±0.34c |

[CNE; Carvacrol nanoemulsion with different concentration of Tween 80 i.e., 2.5, 5, 7.5, and 10 %, (v/v)].

**Table S-3.** Zone of inhibition of coating formulations against selected microbes.

| **Coating formulation code** | **Zone of inhibitions (mm)** | | | |
| --- | --- | --- | --- | --- |
|  | ***Rhizopus stolonifer*** | ***Saccharomyces cerevisiae*** | ***Penicillium italicum*** | ***Aspergillus Niger*** |
| **CF1** | 12.84±0.17a | 13.47±0.01a | 13.15±0.07a | ND |
| **CF2** | 15.06±0.08b | 14.6±0.11b | 13.74±0.20b | 14.35±0.06a |
| **CF3** | 14.03±0.05c | ND | 13.13±0.03a | 13.23±0.05b |
| **CF4** | 14.23±0.17bc | 13.7±0.15a | 14.86±0.03c | 14.26±0.12a |
| **CF5** | 14.6± 0.30bc | 14.21±0.05b | 14.7±0.1c | 14.33±0.13a |

Values are means ± standard deviations of triplicate determinations and the superscript letters indicate that they are significantly different (p < 0.05). [CF1 (3 % carnauba wax); CF2 (3 % shellac wax); CF3 (3 % carnauba wax +3 % shellac); CF4 (3% carnauba wax + 3 % shellac + 2 % CNE-3); and CF5 (3 % carnauba wax +3 % shellac + 4 % CNE-3).]

**Table S-4.** Percent decay index of the mandarin oranges stored for 30 days under ambient conditions

| **Storage period** | **Treatments** | | | | | |
| --- | --- | --- | --- | --- | --- | --- |
|  | **Control** | **CF1** | **CF2** | **CF3** | **CF4** | **CF5** |
| **Day 15** | 2.83±0.16^a^ | ND | ND | ND | ND | 1.33±0.16^b^ |
| **Day 20** | 3.83±0.28^a^ | 1.66±0.28^bc^ | 1.16±0.28^bc^ | 0.83±0.28^c^ | 0.67±0.57^c^ | 2±0.5^b^ |
| **Day 25** | 4.83±0.28^a^ | 3±0.5^b^ | 2.67±0.57^b^ | 2.33±0.57^bc^ | 1.17±0.28^c^ | 3.5±0.5^b^ |
| **Day 30** | 4.96±0.05^a^ | 4.83±0.28^a^ | 3.66±0.57^a^ | 4.33±0.57^a^ | 1.33±0.57^b^ | 4.66±0.57^a^ |

ND: Is represented decay index not detected. Values are means ± standard deviations of triplicate determinations and the superscript letters indicate that they are significantly different (p < 0.05). [Control (uncoated); CF1 (3 % carnauba wax); CF2 (3 % shellac wax); CF3 (3 % carnauba +3 % shellac); CF4 (3 % carnauba + 3 % shellac + 2 % CNE-3); and CF5 (3 % carnauba +3 % shellac + 4 % CNE-3).]
